# Exploring Key Factors Influencing Nursing Students’ Cognitive Load and Willingness to Serve Older Adults: Cross-sectional Descriptive Correlational Study

**DOI:** 10.2196/43203

**Published:** 2023-01-04

**Authors:** Pei-Lun Hsieh, Yu-Rung Wang, Tien-Chi Huang

**Affiliations:** 1 Department of Nursing National Taichung University of Science and Technology Taichung Taiwan; 2 Department of Nursing, Chang Gung University of Science and Technology Chiayi Taiwan; 3 Department of Information Management National Taichung University of Science and Technology Taichung Taiwan

**Keywords:** immersive virtual reality learning, VR learning, mental effort, mental load, service willingness, older adult, virtual reality, nursing student, professional education, digital learning, older adult population

## Abstract

**Background:**

Virtual learning environments (VLEs) use a virtual environment to support learning activities. VLEs are commonly used to overcome the temporal and spatial restrictions of learning activities held in conventional face-to-face classrooms. In VLEs, students can participate in learning activities using the internet, and teachers can provide assistive learning tools during the process.

**Objective:**

The purpose of this study was to investigate the relationships among nursing students’ mental load, cognitive load, and affective learning outcomes in terms of their willingness to serve older adults in an interaction-based educational virtual reality (VR) learning environment.

**Methods:**

This study used a cross-sectional method. A total of 130 students participated in interaction-based VR learning and completed related questionnaires. Descriptive and inferential statistics and stepwise regression for data analysis were used.

**Results:**

The research results revealed that in the dimension of willingness to use VR learning materials, perceived usefulness received the highest score (mean 4.42, SD 0.45). In the dimension of nursing ability, students scored the highest in information management and application ability to care for case patients (mean 4.35, SD 0.54). Correlation analysis revealed that cognitive load during learning and willingness to serve older adults were negatively correlated, whereas willingness to use VR learning materials was positively correlated with nursing ability and willingness to serve older adults. Analyzing the regression coefficients of predictor variables revealed that willingness to use VR learning materials (*β*=.23; *t*_2_=2.89, *P*=.005) and cognitive load during learning (*β*=–.35; *t*_2_=–.4.30, *P*<.001) were predictive factors of nursing students’ willingness to serve older adults.

**Conclusions:**

This study demonstrated that students’ willingness to use VR learning materials and their cognitive load during learning affected their willingness to care for older adults. Therefore, the components of mental or cognitive load generate inconsistent predictive effects on affective variables and willingness to serve older adults.

## Introduction

As a consequence of population aging in many countries, nurses must be equipped with the ability to care for older adults, perform early assessments, and establish care demands. In nurse cultivation and education, technology learning elements are incorporated. Virtual learning environments (VLEs) use a virtual environment to support learning activities. VLEs are commonly used to overcome the temporal and spatial restrictions of learning activities held in conventional face-to-face classrooms. VLEs are learning environments free from temporal and spatial constraints that use a web-based virtual space to ensure representational fidelity and facilitate learner interactions. VLEs are typically established on servers.

In a VLE scenario, learners can perceive a construction of identity and a sense of presence and copresence. VLEs provide learners with multimedia materials and can be paired with teaching strategies. According to teaching goals, models can be designed to provide learning activities that assist learners in completing learning tasks [1,2]. VLEs have the following characteristics. (1) VLEs are planned information spaces. (2) VLEs are social spaces in which teachers and students can interact through teaching and learning processes. (3) Information and social spaces can be presented using text and 2D or 3D virtual reality (VR) modes. (4) In a VLE, students can participate in learning activities and discuss the content of the virtual learning space with teachers. (5) VLEs can integrate various techniques and teaching methods to innovate teaching. (6) VLEs can be used in distance teaching or in reinforcing classroom-based learning activities [1-5].

With advancing technology, content that cannot be presented using conventional teaching methods has gradually been converted using VR capabilities to enhance student learning. VR offer students the virtual experience of being in an actual environment and is suitable for assisting learning for scholars, students, nursing personnel, and patients [6]. In VLEs, students can participate in learning activities using the internet. During the process, teachers can provide assistive learning tools, such as web-based exchange and discussion, web-based tests, uploaded files, assignment submission, peer discussion, peer assessment, grading, questionnaires, and learning-history tracking, thereby generating rich interactive learning experiences [1,2,7].

Hodgson et al [8] used interactive VR lesson plans to support discussion and teaching, discovering that 65% of study participants reported that using VR for case discussion could increase their learning interests and assist them in preparing for future clinical internship scenarios. For the field of nursing, Chiou et al [9] established a VR learning platform to simulate the clinical scenario medication administration. They stated that students exhibited interest in the learning process and improved self-learning efficacy. Ball [10] applied the VR 360 Photosphere environmental platform experimentally, determining its ability to reduce students’ anxiety about actual clinical environments. Herault et al [11] used 360-degree VR videos to simulate patients with trauma for the teaching of nursing students. The videos were divided into topics such as introducing the medical team, admitting patients into the emergency department, inspecting patients, and monitoring patients’ conditions after they have been stabilized. These 4 scenarios supported the learning of students and medical and nursing personnel. Using the number of interactions noted during video coding, the duration of viewing a video, and the questions and answers raised on the basis of a video, the researchers discovered that the students exhibited significant differences in their cognition and learning processes. Hodgson et al [8] developed VR videos on the basis of real-world cases in hospital settings. The VR scenarios involved the interaction between patients and the medical team. Students were immersed in an environment to which they did not usually have access, enabling learning ahead of time. The research results indicated that 65% of the participants reported that discussing VR-based cases increased their learning interests and prepared them for clinical work.

Through a scoping review, Fealy et al [12] explored the effect of using immersive VR in nursing and midwifery education and discovered that using VR for simulated scenario learning provided students with the opportunity for repeated learning, which enhanced students’ care ability and confidence. Foronda et al [6] and Shorey and Ng [13] conducted systematic reviews to investigate the effect of virtual simulation teaching on nursing students, nurses, and nursing education. The results revealed that VR for simulated scenario learning could replace or supplement traditional nursing education, with the VR-based method effectively increasing students’ cognition and knowledge, skills and performance, critical thinking, self-confidence, and learner satisfaction.

Students can learn from VR simulation scenarios that enable interaction. In this study, we conducted a literature review of domestic and foreign studies and discovered that few studies have explored the use of interactive VR learning and its effect on nursing students’ nursing ability and willingness to serve older adults. This study explored self-evaluated nursing ability and willingness to care for older adults in nursing students after they used interactive VR to learn; the influencing factors were also examined. The results can be used to enhance nursing students’ nursing ability in terms of older adult care.

The research questions (RQs) of the study are as follows. RQ1: What are nursing students’ perceptions of their adoption of interaction-based VR learning? RQ2: What are the nursing students’ perceptions of factors (eg, willingness to use VR learning materials for older adult assessment, cognitive load, and willingness to serve for older adult care) that affect the adoption of VR educational technologies?

## Methods

### Research Design and Research Participants

This study adopted a cross-sectional correlation research design. A science and technology university in Taiwan acted as the recruitment site. Purposive sampling was used, and fourth-year students in the nursing department of a 5-year program were recruited as research participants, with those who had suspended their studies excluded. After the participants interacted with the VR-based older adult care assessment system, they completed a questionnaire survey. G*Power software (version 3.1.9.2; Heinrich-Heine-Universität Düsseldorf) was used to estimate the number of research samples. We set the statistical power as .8, type I error (α) as .05, and effect size as .3 (moderate effect). We identified 8 independent variables in this study. The calculation results indicated that the study required a sample size of 121; a total of 130 nursing students were recruited.

**Figure 1:**
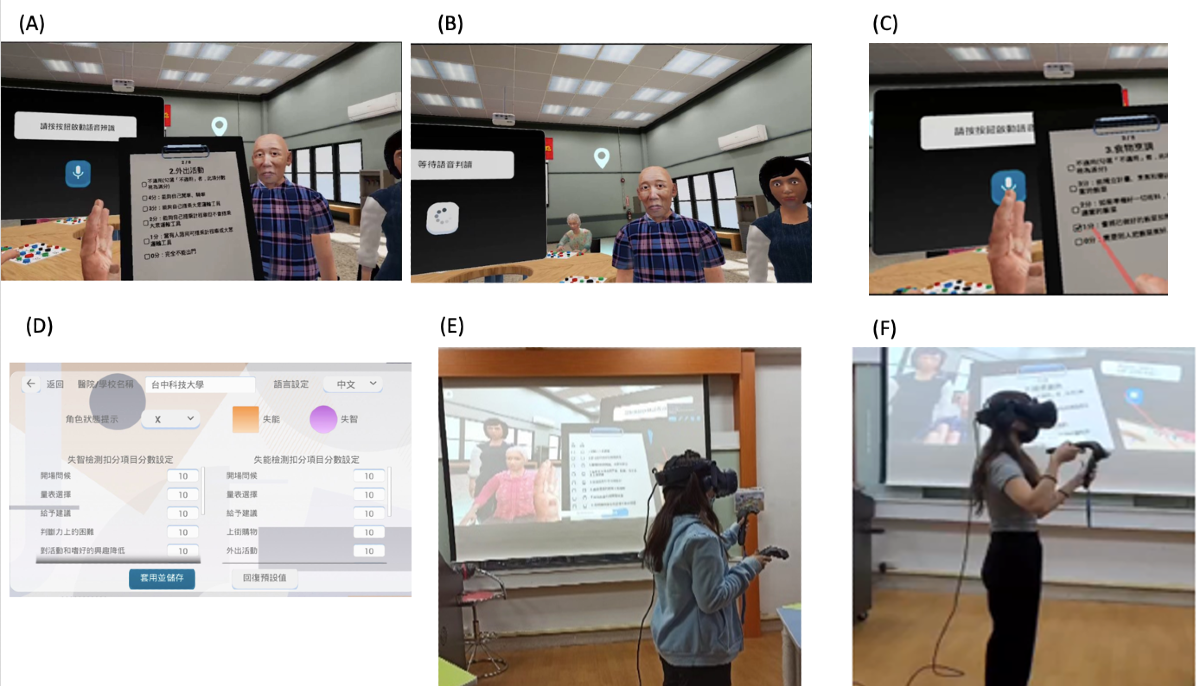
Virtual reality (VR) scenario. (A) Selecting a seated male or female older adult. (B) A caregiver was seated at the older adult’s side. The caregiver could assist in answering questions that the older adult could not answer. (C) The assessment checklist was displayed as a virtual image; users could check the assessment items. (D) Backstage scoring system. Each assessment item (eg, Instrumental Activities of Daily Living scale items including ability to use telephone, shopping, food preparation, housekeeping, laundry, mode of transportation, responsibility for own medications, and ability to handle finances) was scored; the full score was 100. (E) and (F) Nursing students engaging in VR learning.

The research participants were enrolled in an older adult nursing course. In addition to classroom learning, they were instructed to use interactive VR lessons to experience older adult care. The VR lesson plan consisted of 2 units, namely the Ascertain Dementia-8 assessment and Instrumental Activities of Daily Living scale (IADLs). In the VR scenario, the students could interact with several older adults. The students could choose suitable assessment scales according to the older adults’ conditions. For example, if an older adult or primary caregiver reported a decline in the older adult’s memory function, then the student could select the Ascertain Dementia-8 assessment to assess the presence of early-onset dementia. If an older adult had reduced activity functions, then the IADLs could be selected. Using voice recognition and a VR images scale, the students could check the correct answers to scenario-based questions. A full test score of 100 was possible, with 60 representing the passing score. The detailed VR operation process is illustrated in Figure 1.

### Research Instruments

An anonymous structured questionnaire was used for data collection. All research instruments were applied under the developer’s consent. The scales were described as follows.

#### Personal Attributes and Demographic Information

We composed this part of the questionnaire through referencing the literature. Personal attributes consisted of age, sex, education level, and experiences with using VR to learn.

#### Willingness to Use VR Learning Materials for Older Adult Care and Assessment

We referenced the scale based on the technology acceptance model developed by Davis and Venkatesh [14], revising this scale to consist of perceived usefulness (6 items), perceived ease of use (3 items), attitude intention to use technologies (3 items), and perceived flexibility (3 items) regarding self-assessed VR-based older adult care learning materials. A 5-point Likert scale was used for assessment, comprising the options of *strongly disagree*, *disagree*, *neutral*, *agree*, and *strongly agree*.

#### Cognitive Load Scale for Using VR-Based Learning Materials for Older Adult Care and Assessment

Referencing Hwang et al [15] and Paas et al [16], we developed a cognitive load scale consisting of 2 dimensions, namely mental load and mental effort. Mental load was used to assess the perceived burden generated for nursing students when they complete the VR-based older adult care learning tasks. Mental effort was applied to evaluate the degree of cognition and amount of resources required by the nursing students when they complete the VR-based older adult care learning tasks, thereby facilitating the measurement of the perceived difficulty of the learning materials and tasks, the formats in which learning materials were presented, and the students’ feelings about the explanation methods. A 5-point Likert scale was used for assessment, comprising the options of *strongly disagree*, *disagree*, *neutral*, *agree*, and *strongly agree*.

#### Nursing Students’ Service Willingness Scale Regarding Older Adult Care

For the nursing students who used the VR lesson to learn to assess older adults, we used a scale to assess their willingness to serve older adults. The questionnaire design referenced that developed by Hsieh et al [17]. This willingness to serve older adults scale comprised 15 items rated on a 5-point Likert scale, ranging from *strongly disagree* to *strongly agree*; the higher the score, the greater the student’s willingness to serve older adults. All questionnaire items were direct items. Experts were invited to modify the items to ensure construct validity.

### Questionnaire Reliability and Validity

#### Validity

To ensure the content validity and expert validity of the questionnaire, we invited 3 nursing professors in departments related to older adult care and 2 industry experts to review the adequacy of the content of each scale in the questionnaire. We referenced Polit and Beck [18], who suggested a calculation method for content validity indicators in which items scored higher than 3 by experts are retained. After calculating the percentage, we determined that the content validity index of the willingness to use VR learning materials for older adult care and assessment was .98, that of the cognitive load scale for using VR-based older adult care learning materials was .96, and that of the nursing students’ willingness to serve older adults scale was .99. Each scale exhibited favorable content validity.

#### Reliability

Cronbach α coefficient analysis was applied. The Cronbach α of the willingness to use VR learning materials for older adult care and assessment was .96, that of the cognitive load scale was .98, and that of the willingness to serve scale was .97. All scales exhibited favorable internal consistency.

### Ethics Approval

Ethical approval for the study was obtained from the Human Research Ethics Council (CRREC-110-088). The researcher explained the research purpose and process to the participants. To ensure the protection of their personal rights, written informed consent was obtained prior to data collection.

### Data Processing and Analysis

This study used the SPSS statistical software (version 23.0; IBM Corp) for data entry and statistical analysis. A *P* value of <.05 indicated statistical significance. Descriptive statistics involved using the percentage, mean, and SD to present distributions. The independent sample 2-tailed *t* test, one-way ANOVA, Pearson correlation coefficient, and stepwise regression were used for inferential statistics and analysis.

## Results

### Descriptive Variables of Research Participants

Of the 130 participants of this study, most (n=126, 96.9%) were women. Most of the participants were aged 19 years (n=66, 50.8%), followed by those aged 18 (n=62, 47.7%) and 20 (n=2, 1.5%) years. A total of 93.9% (n=122) of the participants had previously used VR, but none had used VR learning materials related to the care of older adults.

### Participants’ Use of VR-Based Learning Materials for Older Adult Care and Assessment and Their Nursing Ability

Regarding the overall research variables, the total mean score of cognitive load during learning was 2.49, that of willingness to use VR learning materials was 4.23, that of nursing ability was 4.25, that of willingness to serve older adults was 3.66, and that of the satisfaction toward the VR learning model was 4.14. The detailed data are presented in Table 1.

**Table 1:** Participants’ use of virtual reality (VR) learning materials to assess older adult care and their nursing ability (N=130).

Research variable, secondary dimension		Secondary dimension, mean (SD)	Total, mean (SD)
**Cognitive load during learning**			2.49 (0.73)
	Mental load	2.45 (0.74)	N/A^a^
	Mental effort	2.52 (0.78)	N/A
**Willingness to use VR learning materials**			4.23 (0.47)
	Perceived usefulness	4.42 (0.45)	N/A
	Perceived ease of use	4.25 (0.61)	N/A
	Willingness to use	3.96 (0.74)	N/A
	Flexibility	4.29 (0.49)	N/A
**Nursing ability**			4.25 (0.48)
	Basic and general educational competencies	4.32 (0.50)	N/A
	Information management and application ability of nursing care case scenario	4.35 (0.54)	N/A
	Health care policies, finance, and environmental capability	4.28 (0.62)	N/A
	Care quality, case content safety, and leadership ability	4.18 (0.70)	N/A
	Nursing practice ability	4.26 (0.49)	N/A
	Preventive health care ability	4.22 (0.54)	N/A
	Teamwork ability	4.09 (0.72)	N/A
	Risk and quality management ability	3.26 (0.75)	N/A
	Ethical ability	3.38 (0.65)	N/A
Willingness to serve older adults		N/A	3.66 (0.40)
Satisfaction toward the VR learning model		N/A	4.14 (0.52)

^a^N/A: not applicable.

### Difference Analysis of Demographic Data and Research Variables

The independent sample 2-tailed *t* test was used to explore whether sex-based differences were present among the 4 variables of cognitive load during learning, willingness to use VR learning materials, nursing ability, and willingness to serve older adults; Levene test with equal variance was used to obtain the *F* test values of the 4 aforementioned variables, which were 4.13, .01, .34, and 5.07, respectively. Among them, cognitive load during learning and willingness to serve older adults had *P* values of <.05. Next, the *t* value and significance were used for data analysis. The *t* values of the 4 variables of cognitive load during learning, willingness to use VR learning materials, nursing ability, and willingness to serve older adults were .92, 1.52, 1.23, and –14.53, respectively. Only the *t* value of willingness to serve older adults exhibited a significant difference (*P*<.001). According to the mean values listed in Table 2, women’s willingness to serve older adults was significantly higher than that of men (*P*<.001).

**Table 2:** Sex-based differences in research variables (N=130).

Dimension	Participants, n		Research variable, mean (SD)			*F* test		*t* test	
	Male	Female	Male	Female	*P* value	Value	*P* value	Value (*df*)	*P* value
Cognitive load during learning	4	126	3.00 (1.15)	2.47 (0.71)	.43	4.13	.04	.92 (128,3)	.43
Willingness to use VR^a^ learning materials	4	126	4.58 (0.48)	4.22 (0.47)	.13	.01	.91	1.52 (128,3)	.13
Nursing ability	4	126	4.53 (0.54)	4.24 (0.47)	.22	.34	.56	1.23 (128,3)	.22
Willingness to serve older adults	4	126	3.10 (0.04)	3.68 (0.39)	<.001	5.07	.03	–14.53 (128,43)	<.001

^a^VR: virtual reality.

### Correlation Analysis of Cognitive Load During Learning, Willingness to Use VR Learning Material, Nursing Ability, and Willingness to Serve Older Adults

According to the analysis results summarized in Table 3, the correlation coefficient of cognitive load during learning and willingness to serve older adults was –.374 (*P*<.001), indicating that these 2 variables were significantly and negatively correlated. The correlation coefficient of willingness to use VR learning materials and nursing ability was .693 (*P*<.001), indicating that these 2 variables were significantly and positively correlated. The correlation coefficient of willingness to use VR learning materials and willingness to serve older adults was .274 (*P*<.001), indicating that these 2 variables were significantly and positively correlated.

**Table 3:** Correlation analysis.

Research variable		Cognitive load during learning	Willingness to use VR^a^ learning materials	Nursing ability	Willingness to serve older adults
**Cognitive load during learning**					
	*r*	1	—^b^	—	—
	*P* value	—	—	—	—
**Willingness to use VR learning materials**					
	*r*	–.122	1	—	—
	*P* value	>.05	—	—	—
**Nursing ability**					
	*r*	–.006	.693	1	—
	*P* value	.94	<.001	—	—
**Willingness to serve older adults**					
	*r*	–.374	.274	.138	1
	*P* value	<.001	<.001	>.05	—

^a^VR: virtual reality.

^b^Not applicable.

### Regression Analysis of Willingness to Use VR Learning Materials and Cognitive Load During Learning on Willingness to Serve Older Adults

We applied regression analysis, and the model variance analysis results in Table 4 indicated that the overall regression model reached a significant level (*F*_2_=15.133, *P*<.001), indicating that the overall regression model was statistically meaningful. After adjustment, the coefficient of determination was .180. Following the *F* test, we conducted an analysis of the regression coefficients of the predictor variables. The results revealed that the standardized regression coefficient (*β*) of willingness to use VR learning materials was .23 (*t*_2_=2.89, *P*=.005), reaching a significant level; the *β* of cognitive load during learning was −.35 (*t*_2_=–4.30, *P*<.001), also reaching a significant level. The standardized regression coefficient represented the amount of variance explained by the individual predictor variables for the dependent variables.

**Table 4:** Regression analysis of willingness to use virtual reality (VR) learning materials and cognitive load during learning on willingness to serve older adults. The adjusted *R*^2^ was 0.18 (*F*_2_=15.13, *P*<.001).

Model	Unstandardized coefficient, B estimation value (SE)	Standardized coefficient beta distribution	*t* test (*df*)	*P* value
Constant	3.30 (.32)	.32	10.36 (2,127)	<.001
Willingness to use VR learning materials	.19 (.07)	.23	2.89 (2,127)	.005
Cognitive load during learning	–.19 (.04)	–.35	–4.30 (2,127)	<.001

## Discussion

### Participants’ Use of VR Learning Materials for Assessing Older Adult Care and Nursing Ability Under Personal Attributes

Among the research participants, 96.9% were women, 50.8% were aged 19 years, and 93.9% had previously used VR but not VR learning materials related to the care of older adults. This result indicates that VR has been widely applied in daily life but little older adult care learning materials had incorporated VR, resulting in few opportunities for students to learn and experience this immersive technology. The overall learning satisfaction of the students using VR learning materials to assess older adult care was high, a result that is in line with those of the systematic literature review of Gasteiger et al [19].

Under the dimension of willingness to use VR learning materials, perceived usefulness scored the highest among all aspects. This result is similar to that of related studies [20-22], indicating that using VR to assist learning reinforced the cognitive level of learners and assisted them in their learning. Regarding the nursing ability dimension, information management and application ability to care for case patients scored the highest among all aspects, indicating that through engaging with the interactive VR scenario, learners’ ability to perceive and respond to scenario-based problems was enhanced. The participants could use voice recognition to respond and select the correct care method. The learning process was valuable for the participants’ future case management ability, a result echoing those of other studies [22,23].

### Significant Correlation Factors of Cognitive Load During Learning, Willingness to Use VR Learning Materials, Nursing Ability, and Willingness to Serve Older Adults

This study revealed sex-based differences in the participants’ willingness to serve older adults, with women being significantly more willing to do so than did men. This result may be attributable to the nursing department being composed largely of women, and thus their willingness to care was higher than that of men. This result is in line with that of Guo et al [24].

The mean score of cognitive load during learning in this study was low, reflecting that during the research process, the older adult care assessment learning materials were not perceived as difficult or burdensome by the learners. If participants can be equipped with relevant older adult care knowledge in the classroom before experiencing VR, their cognitive load during learning can be reduced. Notably, cognitive load during learning and willingness to serve older adults were negatively correlated. This result indicated that when teachers choose learning materials, in addition to making selections according to the teaching objectives and content of the professional course, they must also consider the difficulty and appropriateness of the content. During the students’ learning process, teachers must adjust the course content according to the learners’ cognitive load, thereby increasing learners’ learning effectiveness and their willingness to care for older adults. This finding is consistent with those of other studies [17,25,26].

Willingness to use VR learning materials and nursing ability were positively correlated, demonstrating the essentiality of the vividness of the learning environment. Through the use of VR to present a vivid scenario of caring for older adults, the nursing students’ ability to provide professional nursing care to older adults was enhanced. From the positive feedback scenario of interactive VR, the opportunities for nursing students to interact with older adults were increased, which reinforced the students’ willingness to serve older adults. This research result is in line with those of related studies [21,27-30].

### Critical Factors Influencing Willingness to Serve Older Adults

This study discovered that willingness to use VR learning materials and cognitive load during learning were critical factors influencing willingness to serve older adults. This result indicated that engaging in the interactive VR scenario increased the students’ willingness to use such materials. Additionally, with VR supporting conventional classroom teaching, students may repeatedly practice older adult care scenarios, allowing them to become familiar with the relevant knowledge on a cognitive level. They can engage in learning without worrying about making mistakes and harming older adults. Thus, students’ learning load was reduced, and their problem-solving ability and self-confidence increased. These results are consistent with those of other studies [17,21,27,29-31].

Studies of willingness to serve older adults [10,13,17,24,32,33] have reported that through learning to interact with older adults, nursing students can observe older adults’ verbal and nonverbal cues, with such interaction increasing their willingness to serve and care for older adults. This study used VR learning materials, and the results revealed that through the VR simulation scenario, the opportunities for students to interact with older adults were increased. Learning through VR scenarios can reduce students’ anxiety when communicating with older adults in a real-world context, thereby increasing students’ willingness to serve older adults.

### Limitations

This study had some limitations. The respondents provided subjective responses, which could sometimes obscure their actual intention, and more male participants should have been included in the study. Moreover, this study was a quantitative study, which increased the difficulty of understanding the nursing students’ willingness to serve older adults.

### Conclusions

This survey of 130 preregistered nurses explored factors affecting their willingness to serve older adults. The most influential factors were willingness to use VR learning materials and cognitive load during learning, both of which can be enhanced through older adult care–related courses and practical experience. Nurses play a critical role in the care of older adults, and courses on older adult care are thus crucial for developing sufficient professional human resource capacity. We recommend that VR case scenarios about older adult nursing assessment be incorporated into in-service education. Through repeated practice using these scenarios, nurses’ willingness and ability to care for and serve older adults can increase. Longitudinal follow-up studies of recent nursing graduates (preregistered nurses) who had engaged in VR-based older adult care courses must be conducted to elucidate these courses’ effects on nurses’ older adult care competence and willingness to serve older adults.

## Abbreviations

IADLs: Instrumental Activities of Daily Living scale
RQ: research question
VLE: virtual learning environment
VR: virtual reality
